# Influence of supply-side factors on voluntary medical male circumcision costs in Kenya, Rwanda, South Africa, and Zambia

**DOI:** 10.1371/journal.pone.0203121

**Published:** 2018-09-13

**Authors:** Sergio Bautista-Arredondo, Sandra G. Sosa-Rubi, Marjorie Opuni, David Contreras-Loya, Gina La Hera-Fuentes, Ada Kwan, Claire Chaumont, Abson Chompolola, Jeanine Condo, Kumbutso Dzekedzeke, Omar Galarraga, Neil Martinson, Felix Masiye, Sabin Nsanzimana, Richard Wamai, Joseph Wang’ombe, on behalf of the ORPHEA study team

**Affiliations:** 1 Division of Health Economics and Health Systems Innovations, National Institute of Public Health (INSP), Cuernavaca, Mexico; 2 School of Public Health, University of California, Berkeley, United States of America; 3 Independent Consultant, Geneva, Switzerland; 4 T.H. Chan School of Public Health, Harvard University, Boston, United States of America; 5 Division of Economics, University of Zambia, Lusaka, Zambia; 6 School of Public Health, National University of Rwanda, Kigali, Rwanda; 7 Dzekdzeke Research & Consultancy, Lusaka, Zambia; 8 School of Public Health, Brown University, Providence, United States of America; 9 Perinatal HIV Research Unit, University of the Witwatersrand, Johannesburg, South Africa; 10 Rwanda Biomedical Center, Kigali, Rwanda; 11 College of Social Science and Humanities, Northeastern University, Boston, United States of America; 12 School of Public Health, University of Nairobi, Nairobi, Kenya; Universidad Loyola Andalucia, SPAIN

## Abstract

**Background:**

In this study, we described facility-level voluntary medical male circumcision (VMMC) *unit cost*, examined *unit cost* variation across facilities, and investigated key facility characteristics associated with *unit cost* variation.

**Methods:**

We used data from 107 facilities in Kenya, Rwanda, South Africa, and Zambia covering 2011 or 2012. We used micro-costing to estimate economic costs from the service provider’s perspective. Average annual costs per client were estimated in 2013 United States dollars (US$). Econometric analysis was used to explore the relationship between VMMC *total* and *unit cost* and facility characteristics.

**Results:**

Average VMMC *unit cost* ranged from US$66 (SD US$79) in Kenya to US$160 (SD US$144) in South Africa. *Total cost* function estimates were consistent with economies of scale and scope. We found a negative association between the number of VMMC clients and VMMC *unit cost* with a 3% decrease in *unit cost* for every 10% increase in number of clients and we found a negative association between the provision of other HIV services and VMMC *unit cost*. Also, VMMC *unit cost* was lower in primary health care facilities than in hospitals, and lower in facilities implementing task shifting.

**Conclusions:**

Substantial efficiency gains could be made in VMMC service delivery in all countries. Options to increase efficiency of VMMC programs in the short term include focusing service provision in high yield sites when demand is high, focusing on task shifting, and taking advantage of efficiencies created by integrating HIV services. In the longer term, reductions in VMMC *unit cost* are likely by increasing the volume of clients at facilities by implementing effective demand generation activities.

## Background

Voluntary medical male circumcision (VMMC) is an effective [1–3] and cost-effective [4–6] intervention to reduce heterosexual acquisition of HIV by men that is recommended in countries with high HIV prevalence and low levels of male circumcision [7]. Fourteen countries in sub-Saharan Africa (SSA)–including Kenya, Rwanda, South Africa, and Zambia–are scaling up service delivery of adult VMMC for HIV prevention [8].

In the context of plateauing global resources for HIV services [9], policy makers and implementers require information on how to ensure that quality VMMC services are delivered at the lowest cost. To identify opportunities to increase efficiency and plan adequately, they need to understand the ways in which VMMC service delivery costs vary across facilities as well as the factors associated with lower and higher costs that can be acted upon to reduce cost.

There are a growing number of multi-facility costing studies on adult VMMC in the peer-reviewed literature [10–14]. However, most have been conducted within one country comparing *unit cost* across service delivery models [11–14]. Few studies have measured VMMC *unit cost* across relatively large samples of service delivery sites [10, 12], or used econometric analysis to assess cost drivers and possible efficiency gains in VMMC service delivery [10].

In this analysis, we described the average cost per VMMC client in 107 facilities in Kenya, Rwanda, South Africa, and Zambia, examined *unit cost* variation across facilities, and investigated key facility-level characteristics associated with cost variation using an econometric approach. We used data from the *Optimizing the Response in Prevention*: *HIV Efficiency in Africa* (ORPHEA) study [15, 16]–a cross-sectional, micro-costing study conducted between 2012 and 2013, which collected year-long data for either 2011 or 2012.

## Methods

### Study sample

As described previously [15, 16], Kenya, Rwanda, South Africa, and Zambia were purposively selected to reflect cross-country variation in HIV burden and HIV prevention intervention coverage levels; and multistage sampling was used to select sub-national areas within each country and then randomly select health facilities providing VMMC services alone or in combination with HIV testing and counseling (HTC) and/or prevention of mother-to-child transmission (PMTCT) (S1 Fig).

### Description of services

We assessed the costs of facility-based VMMC services. Though VMMC services targeted males aged 15–49 years, services were also provided to males outside this age range–especially younger males. Because of limitations in data on VMMC client age, costs were estimated for all VMMCs provided in sampled facilities. While non-surgical devices (Pre-Pex and Shang Ring) were piloted in Rwanda at the time of the study, the vast majority of circumcisions in all countries were surgical. We assessed routine VMMC services offered consistently year-round as well as intermittent services provided in facilities during high volume campaigns [17]. The following VMMC features were assessed: VMMC counseling, HIV testing, medical examination, and surgical circumcision.

### Data collection

As described in detail elsewhere [15, 16], the ORPHEA study used standardized survey tools to collect information from the perspective of service providers comparable across facilities and countries. Data collection was staggered by country from October 2012 to December 2013 and we collected information retrospectively by month for the calendar year prior to data collection (2011 or 2012). We used micro-costing methods in which quantity and price of inputs were gathered along with information on outputs or services provided; and we adopted an economic costing perspective collecting data irrespective of funding source and valuing donated inputs at their opportunity costs determined by local market prices. As explained previously [15, 16], we gathered data on three input cost categories: personnel, recurrent inputs and services, and capital (equipment and vehicles); and two activity cost categories: training and supervision, valued according to opportunity cost of staff time dedicated to these activities. We obtained from facility program records both the total number of circumcisions performed during each month of the costing year and the total number of procedures completed during the costing year. In cases where those two numbers did not match, the larger number was used (see S1 Methods for sensitivity analysis of this decision).

### Cost estimation

Calculation of personnel costs was determined by the seasonal variation in VMMC service delivery (S2 Fig). Because of this seasonality, assessing staff time use on given days and times through time-motion observation [18, 19] would unlikely be representative of the entire year. To calculate annual staff costs for VMMC services, we first derived a measure of staff effort expended on VMMC service delivery by triangulating individual-level information on time use obtained from program records, self-reports, field notes, and interviews with staff in charge of facilities. When providers reported working on VMMC only, all of their time was allocated to VMMC. In the case of non-dedicated providers, effort attributable to VMMC was a facility-level estimate given by the proportion of annual VMMC clients with respect to the total number of outpatient clients in the facility. Annual staff costs were then obtained by multiplying effort by the average number of weeks and hours worked during the year (by provider type category), multiplied by the average annual salary by provider type (see S2 Methods for more detail).

Total annual VMMC costs were calculated for each facility. Staff costs, recurrent inputs and services, capital, training, and supervision were aggregated for the year of observation. For recurrent services, capital, training, and supervision shared with other interventions, we weighted annual costs by the annual number of VMMC clients over the annual number of outpatient clients in the facility, and the median weight was imputed in 11 facilities with no data on outpatient clients. Facility-level average costs per VMMC (*unit costs*) were obtained by dividing health facilities’ total annual VMMC costs by the annual number of male circumcisions performed.

All cost data were converted from local currencies to United States dollars (US$) using mid-year exchange rates for 2011 (Kenya: 88.81 Kenyan shillings and Zambia: 4,860.7 Zambian kwacha) and 2012 (Rwanda: 614.3 Rwandan francs and South Africa: 8.21 South African Rand), and then inflated to 2013 prices. We report both unadjusted costs and costs adjusted for purchasing power parity (PPP).

### Data analysis

We compared the average cost per VMMC client (*unit cost*) across facilities and countries. We assessed cost composition–with a more in-depth appraisal of VMMC staff costs. We also explored the association between average VMMC *unit cost* and supply-side factors previously shown to be associated with the cost of VMMCs [10], other HIV prevention interventions [20–25], and HIV treatment [25–28]. S1 and S2 Tables provide descriptions of each of the factors included in our analysis.

In order to explore evidence of economies of scale and scope and the association between costs and characteristics of service provision, we modeled *total costs* as a function of outputs and other explanatory variables. We used generalized linear model (GLM) estimation, which is a Maximum Likelihood generalization of the ordinary linear regression approach. This method allowed for more flexibility in the assumption of the error variance distribution. We assumed an identity link function and a Gaussian probability distribution, following the results of the modified Park test [29]. The models were estimated as:

$$\ln\left(TC\right)=b_{0}+b_{1}\ln\left(q\right)+b_{2}\ln{\left(q\right)}^{2}+b_{3}\exp+b_{4}exp^{2}+b_{5,1}\mathrm{HTC}+b_{6,2}\mathrm{PMTCT}+b_{7,3}\mathrm{ART}+b_{8}X+e$$ (1)

where *q* represents the number of annual VMMCs; *exp* captures staff experience providing HIV services in years; *HTC* and *PMTCT* measure the log of the annual number of HTC and PMTCT clients, respectively; *ART* is a binary variable indicating whether the facility provided antiretroviral therapy (ART) or not; and *X* is a vector of supply-side indicators such as level of service provision, whether the facility implemented task shifting (the delegation of tasks to less specialized staff), and whether or not the facility performed community outreach. The models controlled for input prices with a facility-specific index of salaries. Other essential input prices such as circumsicion kits do not vary across facilities. We explored the role of scale by estimating three specifications of the model; we began without adjusting for scale and then sequentially added the linear and quadratic terms of the log of VMMC clients. In all specifications, we tested for heteroskedasticity applying the Breusch-Pagan test and applied robust standard errors when appropriate. We also examined the Variance Inflation Factor (VIF) to assess the presence of multicollinearity.

To further explore economies of scale and the direction and magnitude of the association between *unit costs* and characteristics of services, we performed GLM regressions with the natural log of the VMMC *unit cost* as a function of output and supply-side characteristics.

To examine the impact of cost outliers (i.e. high cost facilities with cost per VMMC greater than US$ 400) on our findings, we also ran regressions excluding these high cost facilities.

### Ethical clearance

The ORPHEA study was approved by the ethical review boards at the following institutions: National Institute of Public Health, Mexico; Kenyatta National Hospital and University of Nairobi; Northeastern University in Boston; Rwanda Biomedical Center; University of the Witwatersrand in Johannesburg; and University of Zambia. Written informed consent was obtained from all service providers interviewed in the study.

## Results

The analytic sample and sample characteristics are shown in Table 1. Of the 107 facilities in the sample, 33 were in Kenya, 32 were in Rwanda, 25 were in South Africa, and 17 were in Zambia. Whereas 67 of the facilities were primary care facilities, 40 were hospitals. Sites were on average larger (in terms of annual number of VMMCs performed) in Kenya and South Africa than in Rwanda and Zambia. Key input prices including HIV test kits, circumcision kits, and staff salaries were highest in South Africa and roughly comparable across the other three countries.

**Table 1:** Average annual cost per VMMC client at the facility-level, supply-side factors, and input prices.

	Kenya				Rwanda				South Africa				Zambia				Total			
	(N = 33: 12 H, 21 PHC)				(N = 32: 7 H, 25 PHC)				(N = 26: 17 H, 9 PHC)				(N = 17: 5 H, 12 PHC)				(N = 108: 41 H, 67 PHC)			
	N	Mean	SD	Median	N	Mean	SD	Median	N	Mean	SD	Median	N	Mean	SD	Median	N	Mean	SD	Median
**VMMC COSTS**																				
Average cost per VMMC^a^	33	66	79	42	32	75	112	29	25	160	144	117	17	95	171	37	107	95	127	52
Weighted average^b^ cost per VMMC	33	41	-	-	32	70	-	-	25	104	-	-	17	45	-	-	107	65	-	-
Average cost per VMMC PPP^a^	33	147	193	87	32	145	244	49	25	234	229	152	17	165	311	60	107	170	237	87
Weighted average^b^ cost per VMMC PPP	33	85	-	-	32	135	-	-	25	147	-	-	17	75	-	-	107	113	-	-
**SUPPLY-SIDE FACTORS**																				
Annual VMMCs performed	33	869	798	663	32	342	392	180	25	1720	2448	913	17	470	533	166	107	847	1385	532
Task shifting	33	0.3	-	-	32	0.9	-	-	25	0.4	-	-	17	0.8	-	-	107	0.6	-	-
Community outreach	31	0.7	-	-	30	0.2	-	-	25	0.5	-	-	16	0.8	-	-	107	0.5	-	-
**INPUTS PRICES**																				
HIV test kit	-	0.8	-	-	-	1.1	-	-	-	4.7	-	-	-	0.7	-	-	-	1.8	-	-
Circumcision kit^c^	-	8.5	-	-	-	10.2	-	-	-	18	-	-	-	9	-	-	-	11.4	-	-
Average staff salary per hour^d^	33	2.1	2.1	1.6	32	2.3	1.7	1.7	25	8.0	8.4	5.1	17	2.8	1.3	3.3	107	4.4	5.9	2.7
**OUTPATIENT CLIENTS**																				
Outpatient clients per year	29	14,351	12,281	10,659	32	16,488	13,310	12,535	19	56,057	34,618	56,837	16	25,339	22,851	16,150	96	25,149	25,762	12,968

H = number of hospitals; N = number of VMMC facilities; PHC = number of primary health care facilities; PPP = purchasing power parity; SD = standard deviation; VMMC = voluntary medical male circumcision; Unit costs and prices in 2013 US$.

^a^South Africa mean VMMC cost is statistically different from the rest of the countries (t-test).

^b^Weighted average represents a nationally representative average value, taking into account the relative contribution of each facility in terms of its patient volume. It was calculated as the sum of each data point multiplied by a nonnegative weight (defined as the number of annual VMMC clients/outpatient health clients). Therefore, data points with a higher weight contribute more to the weighted mean than do elements with a low weight.

^c^Disposable circumcision kit includes: container tray, gauzes, syringe and injection needles, gloves, aprons, swabs, needle holder, suture scissor, forceps and clamps and scalpel. Consumables prices were obtained at the national level and thus reflect no inter-facility variation.

^d^Average staff salary per hour of personnel working on VMMC

### Average VMMC unit cost

**Fig 1:**
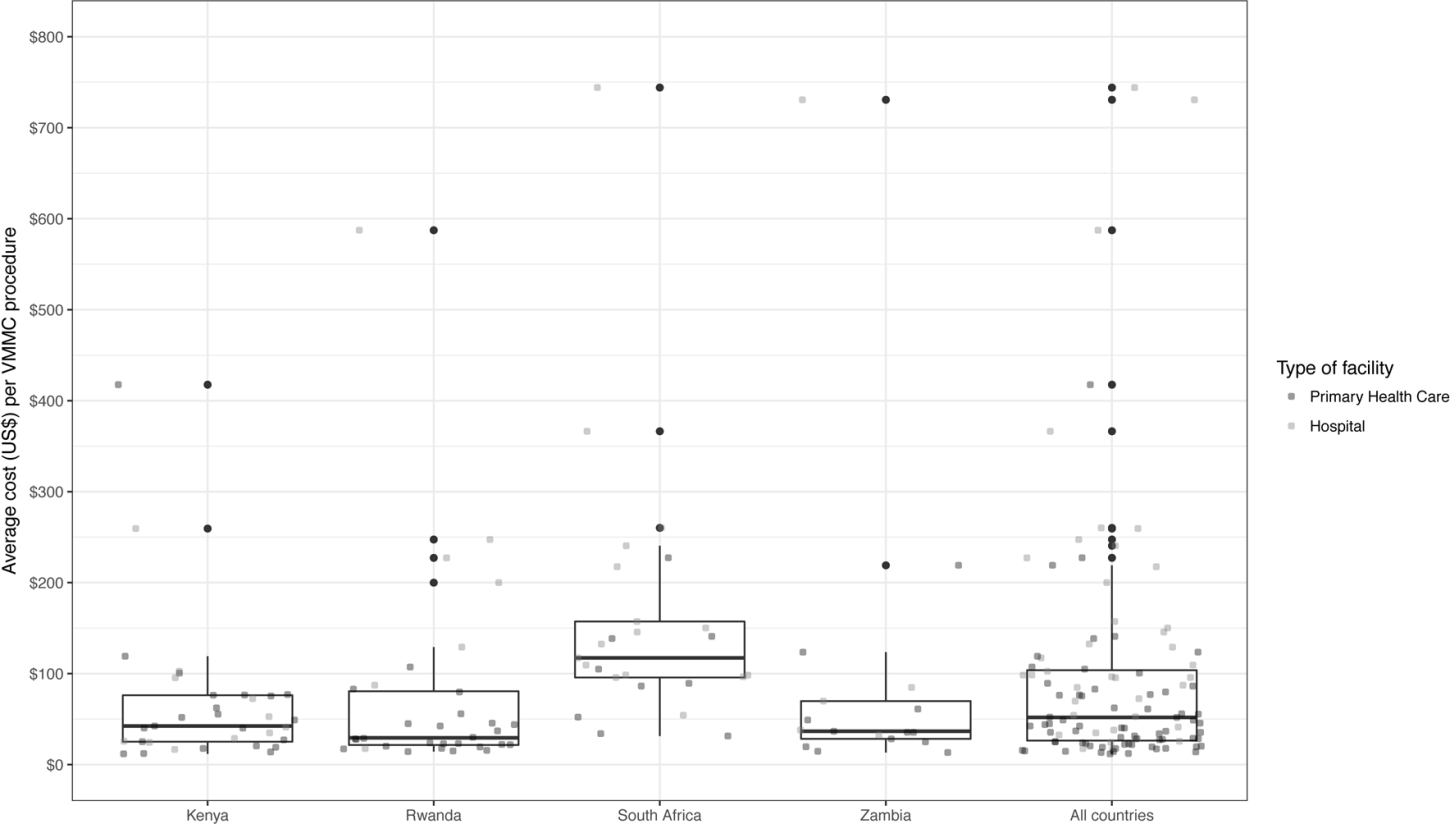
VMMC *unit cost* variation by type of facility and country. Lines inside the box indicate the median of the distribution; boxes depict the inter-quartile range (IQR); whiskers extend to 1.5 times the IQR; one observation was excluded from the plot (>$800US).

Table 1 also presents the average cost per VMMC client in the four countries studied. Average VMMC *unit cost* ranged from US$66 in Kenya to US$160 in South Africa. Table 1 also shows average cost per VMMC client in PPP-adjusted dollars which ranged from US$145 in Rwanda to US$234 in South Africa. After adjusting for differences in purchasing power in the four countries, average cost per VMMC client was not significantly different. Also displayed in Table 1 is the VMMC *unit cost* variation within countries. Median *unit cost* was much lower than average *unit cost* in all countries, revealing a skewed distribution of per client cost with some facilities in each country having very high costs. This within country cost variation is further illustrated in Fig 1, which displays the dispersion in average per client cost by facility type.

### Average VMMC unit cost composition

**Fig 2:**
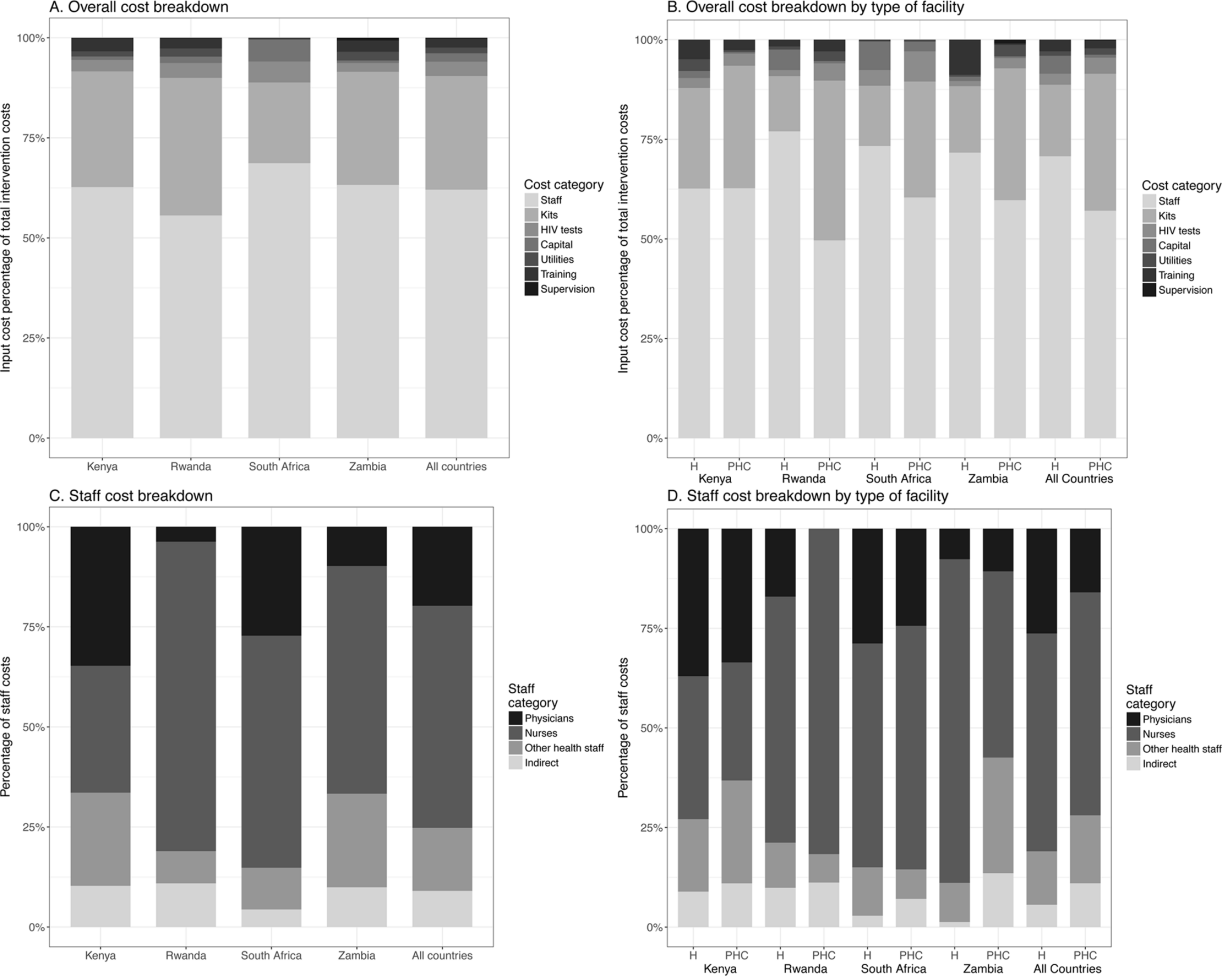
Overall and country-level breakdown of *unit cost* by cost category and breakdown of staff costs by type of staff. H = hospital, PHC = primary health care facilities. General and specialized nurses were included in *nurses* category.

The largest components of VMMC *unit cost* in all countries were staff costs (Fig 2 Panel A)–although they represented smaller proportions in primary care facilities compared to hospitals (Fig 2 Panel B). Circumcision kits and HIV test kits made up the second largest shares. Fig 2 Panels C and D also show the breakdown of costs for staff associated with VMMC service delivery. Nurses dominated the provision of VMMC in Rwanda, South Africa, and Zambia, while physicians played important roles in Kenya and South Africa.

### VMMC costs and supply-side factors

Table 2 shows the results of three specifications of the regression model of the natural log of VMMC *total cost* against supply-side factors. Specification 1 included input prices, basic facility characteristics, staff characteristics, outreach, and a set of variables exploring the influence of integration of VMMC with other HIV services; Specification 2 incorporated scale, measured as the natural log of the annual number of VMMC clients; and Specification 3 added the quadratic term of scale. All specifications included nine observations with imputed values for number of HTC or PMTCT clients, using multivariate imputation methods. All specifications included country-level dummy variables.

**Table 2:** GLM regression models. Dependent variable is the natural logarithm of the facility-level total cost of VMMC services.

	Specification (1)^b^^,^ ^d^	%	Specification (2)^b^	%	Specification (3)^b^	%
Annual number of VMMC clients (ln)			0.657***	7^c^	1.521***	15^c^
			(0.529–0.785)		(0.596–2.445)	
Square of annual number of VMMC clients (ln)					-0.070*	-1^c^
					(-0.144–0.004)	
Primary health care facility ^a^	-0.431*	-35	-0.371**	-31	-0.362**	-30
	(-0.896–0.035)		(-0.689 - -0.053)		(-0.676 - -0.049)	
Average staff experience (in years)	0.229*	26	0.246**	28	0.228**	26
	(-0.015–0.473)		(0.053–0.439)		(0.037–0.420)	
Square of average staff experience (in years)	-0.016*	-2	-0.016**	-2	-0.015**	-1
	(-0.033–0.000)		(-0.030 - -0.002)		(-0.029 - -0.001)	
Outreach	0.329	39	0.278*	32	0.297**	35
	(-0.123–0.781)		(-0.019–0.576)		(0.003–0.591)	
Task shifting	-0.676***	-49	-0.532***	-41	-0.526***	-41
	(-1.040 - -0.313)		(-0.840 - -0.224)		(-0.830 - -0.222)	
Annual number of HTC clients (ln)	-0.025	0^c^	-0.061	-1^c^	-0.046	0^c^
	(-0.137–0.087)		(-0.143–0.021)		(-0.128–0.036)	
Annual number of PMTCT clients (ln)	-0.192***	-2^c^	-0.116***	-1^c^	-0.124***	-1^c^
	(-0.285 - -0.099)		(-0.200 - -0.032)		(-0.208 - -0.041)	
Facility provides ART	-0.827**	-56	-0.682**	-49	-0.641**	-47
	(-1.576 - -0.077)		(-1.204 - -0.159)		(-1.157 - -0.124)	
Constant	11.413***		6.791***		4.135***	
	(10.586–12.240)		(5.647–7.936)		(1.102–7.169)	
Observations	100		100		100	

ARV = antiretroviral: HTC = HIV testing and counseling; PMTCT = prevention of mother-to-child transmission; VMMC = voluntary medical male circumcision. All models are adjusted by country dummies. 95% confidence interval in parentheses.

*** p<0.01

** p<0.05

* p<0.1.

^a^Reference category: Hospital.

^b^Nine observations with missing values on the number of HTC or PMTCT clients were imputed using 90 observations with the linear regression model: HTC (or PMTCT) number of clients = *b*_0_ + *b*_1_staff + *b*_2_VMMC + *b*_3_facility type + *b*_4_country + e. % Percentage change in *total cost* compared to the reference category.

^c^Percentage change in *total cost* per 10% change in independent variable.

^d^GLM with robust standard errors (White-Huber)[30]

Level of service provision was negatively associated with VMMC *total cost*, with cost in primary health care facilities about 30% lower than in hospitals (Specification 3). Staff experience was positively associated with VMMC *total cost– 26% higher total cost per additional average year of staff experience*, with decreasing marginal impact as experience increased. Facilities implementing task-shifting showed about 40% lower costs than facilities not employing the strategy. Facilities conducting VMMC outreach showed up to 35% higher total cost than facilities not conducting outreach. Provision of other HIV services was negatively associated with VMMC *total cost*, consistent with economies of scope in the provision of VMMC when other HIV services were also provided (ART and PMTCT). VMMC *total cost* in facilities providing ART was 50% lower than in facilities not providing treatment. Finally, annual number of VMMC clients was positively associated with VMMC *total cost*–with a 15% increase in *total cost* for every 10% increase in number of clients, decreasing at a rate of 1% times the square of the total number of clients, consistent with economies of scale.

Table 3 presents the results of the same specifications using the *unit cost* of VMMC services as the dependent variable. The results are highly consistent with those shown in Table 2.

**Table 3:** GLM regression models. Dependent variable is the natural logarithm of the facility-level average cost per VMMC (unit costs).

	Specification (1)^b^	%	Specification (2)^b^	%	Specification (3)^b^	%
Annual number of VMMC clients (ln)			-0.343***	-3^c^	0.459	5^c^
			(-0.472 - -0.215)		(-0.460–1.378)	
Square of annual number of VMMC clients (ln)					-0.065*	-1^c^
					(-0.139–0.009)	
Primary health care facility^a^	-0.348*	-29	-0.375**	-31	-0.361**	-30
	(-0.710–0.013)		(-0.692 - -0.058)		(-0.675 - -0.047)	
Average staff experience (in years)	0.263**	30	0.251***	29	0.233**	26
	(0.046–0.480)		(0.061–0.442)		(0.043–0.422)	
Square of average staff experience (in years)	-0.016**	-2	-0.016**	-2	-0.015**	-1
	(-0.032 - -0.000)		(-0.030 - -0.002)		(-0.029 - -0.001)	
Outreach	0.160	17	0.201	22	0.223	25
	(-0.158–0.477)		(-0.078–0.480)		(-0.054–0.500)	
Task shifting	-0.494***	-39	-0.578***	-44	-0.571***	-44
	(-0.839 - -0.149)		(-0.882 - -0.274)		(-0.872 - -0.270)	
Annual number of HTC clients (ln)	-0.083*	-1^c^	-0.064	-1^c^	-0.049	0 ^c^
	(-0.176–0.010)		(-0.146–0.018)		(-0.132–0.034)	
Annual number of PMTCT clients (ln)	-0.076	-1^c^	-0.115***	-1^c^	-0.124***	-1^c^
	(-0.170–0.018)		(-0.199 - -0.031)		(-0.207 - -0.040)	
Facility provides ART	-0.638**	-47	-0.709***	-51	-0.670**	-49
	(-1.233 - -0.042)		(-1.232 - -0.186)		(-1.189 - -0.152)	
Constant	4.584***		6.981***		4.518***	
	(3.800–5.369)		(5.850–8.112)		(1.505–7.530)	
Observations	100		100		100	

ARV = antiretroviral: HTC = HIV testing and counseling; PMTCT = prevention of mother-to-child transmission; VMMC, voluntary medical male circumcision. All models are adjusted by country dummies. 95% confidence interval in parentheses.

*** p<0.01

** p<0.05

* p<0.1

^a^Reference category: Hospital.

^b^Nine observations with missing values on the number of HTC or PMTCT clients were imputed using 90 observations with the linear regression model: HTC (or PMTCT) number of clients = *b*_0_ + *b*_1_staff + *b*_2_VMMC + *b*_3_facility type + *b*_4_country + e. % Percentage change in *unit cost* compared to the reference category.

^c^Percentage change in *unit cost* per 10% change in independent variable.

**Fig 3:**
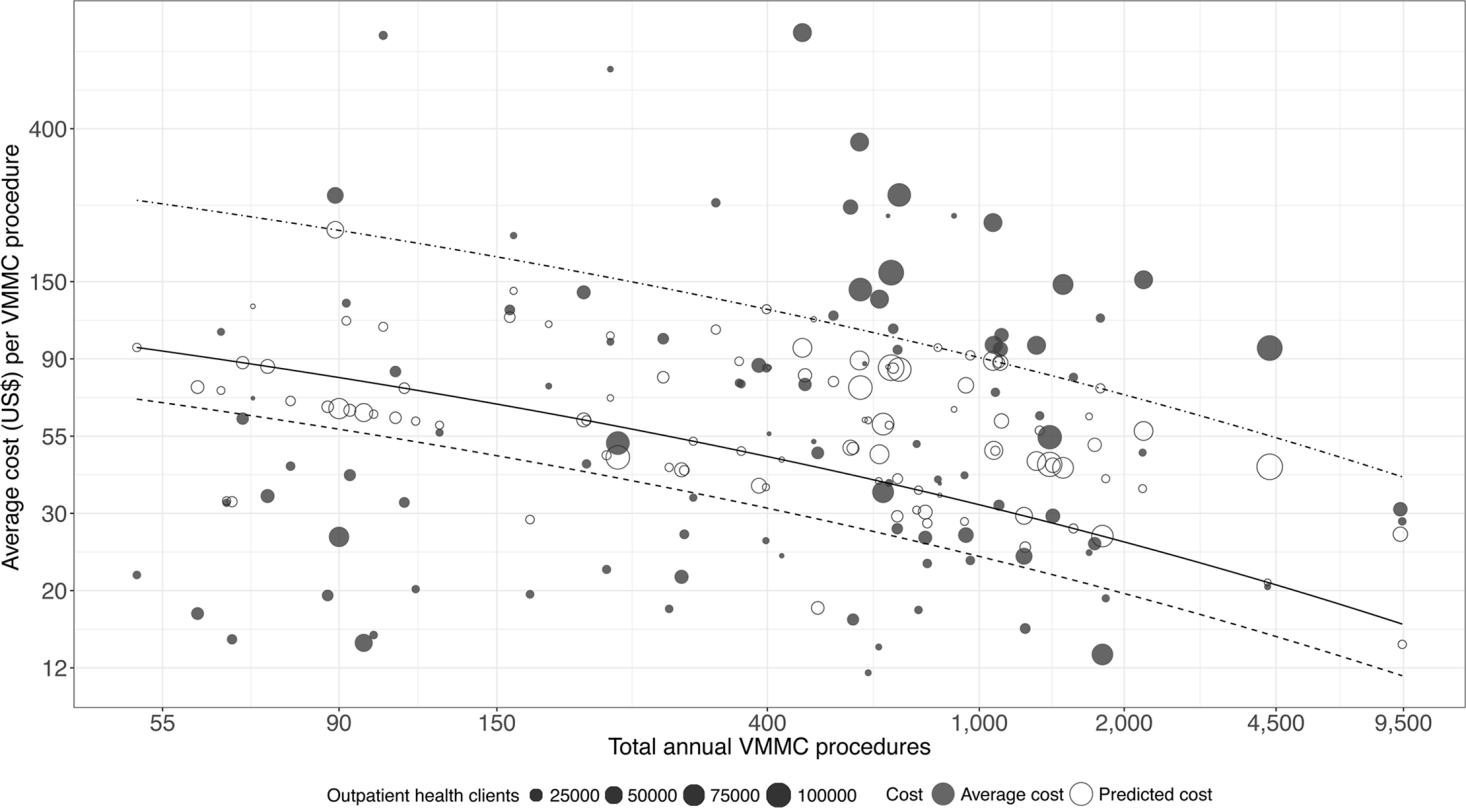
Relationship between scale and average cost per VMMC procedure in three policy scenarios. Solid line = average values of predicted function: Primary health care facility with task shifting, outreach and doesn’t provide ART. Dash-dotted line = Hospital with outreach, without task shifting and doesn’t provide ART. Dashed line = Primary health care facility, with task shifting, outreach and provides ART.

Fig 3 illustrates the effect of select factors on the relationship between scale and VMMC *unit cost*, using the coefficients from Table 3, Specification 2. The solid line depicts the *unit cost* curve with respect to scale, when the values of all variables were fixed at their mean. Fig 3 underscores the importance of economies of scale with *unit cost* ranging from a high of US$98 per circumcision at a scale value of 50 clients per year to around US$16 per circumcision in clinics with around 9,500 clients per year. Fig 3 also shows the *unit cost* curve for two alternative implementation scenarios in which the values of service provision level (primary health care facilities vs. hospitals), task shifting, and ART service provision were fixed at their least and most efficient values (with VMMC outreach included in both scenarios). The *unit cost* maximums and minimums shifted to US$253 and US$42 respectively, in the least efficient scenario; and to US$70 and US$12, respectively, in the most efficient scenario.

Excluding high cost outliers (i.e. high cost facilities with cost per VMMC greater than US$ 400) from our analysis did not alter our findings (S3 and S4 Tables).

## Discussion

Our findings on the variation in VMMC *unit cost* across facilities within and between countries point to substantial inefficiencies in VMMC service delivery. Our results on the facility-level characteristics associated with VMMC *total* and *unit cost* indicate that there are a number of supply-side factors that decision makers can focus on to ensure the delivery of quality VMMC services at the lowest cost. We found evidence of economies of scale and scope in the provision of VMMC services. In addition, we found that whereas staff experience and VMMC outreach were positively associated with VMMC *unit cost*, level of service provision (primary health care facilities vs. hospitals), and the delegation of tasks to less specialized staff (task shifting) were negatively associated with cost per VMMC client.

Our findings on average VMMC *unit cost*–ranging from US$66 in Kenya to US$160 in South Africa–were similar to prior costing study results, although our results for South Africa were higher. Adjusted to 2013 US$ for comparison, average cost per adult VMMC surgery in a fixed site was US$96 in Zambia [10] and ranged from US$41 to US$80 in Kenya [10, 12] and US$102 to US$130 in South Africa [10, 14]. Some of the distinctions between our results and those of previous studies were likely due to differences in costing methods, data collection instruments, and cost elements included. Some of the differences between our findings and earlier work were also likely attributable to distinctions in sampling approaches. Previous micro-costing studies used purposive sampling of facilities. Although our samples were not nationally representative, our study was the first to use systematic sampling to randomly select facilities, thereby including inefficient/costly facilities. Indeed, the median VMMC *unit cost* of US$117 in South Africa was more in line with the *unit costs* found in previous work [10, 14].

Total cost function estimates were consistent with economies of scale and scope. As number of circumcisions (scale) increased, total cost also increased, but at a decreasing rate. In terms of scope, facilities providing ART, PMTCT, or HTC services produced VMMC services at lower total cost, on average, than facilities that did not provide these services.

Our finding on the association between scale and VMMC *unit cost* was consistent with prior studies assessing this relationship [10, 13, 14], as well as earlier work appraising the relationship between scale and other HIV prevention interventions [20–23, 25], and HIV treatment [25–28]. As volume of VMMC clients expands in a facility, reductions in cost per VMMC client are likely as fixed costs are distributed among more clients and staff time is employed more fully. This finding together with our observation on the seasonal variation in VMMC service delivery suggest that one strategy to increase efficiency would be to better exploit existing seasonal variation in demand. At the same time, considerations to scale up VMMC services should include assessments of potential tradeoffs between improved VMMC service access and equity, since focusing exclusively on high-demand times and places would hinder access to VMMC services to some populations. A number of studies have shown that VMMC *unit cost* in mobile services was higher than in fixed sites [11, 13]. We are not aware of cost comparisons between low volume fixed sites and mobile services and such analyses would be important.

Our result on economies of scope offered a new insight on VMMC efficiency. Providing VMMC services in facilities also providing other HIV services could lead to efficiency gains in VMMC service delivery. To our knowledge, this has not been explored previously although it is in line with findings on the cost and efficiency of integration of other HIV services [31].

Our finding that VMMC cost per client in primary health care facilities was lower than in hospitals was consistent with previous work on VMMC [10]. It was also in line with the results of a study assessing the determinants of HIV treatment *unit cost* which found that controlling for patient volume and site maturity, primary-level sites were no more expensive and possibly less expensive than sites at higher levels of the health system [27].

Finally, our finding that staff costs were the most important component of VMMC *unit cost* in all four countries was consistent with prior studies [10, 13, 14]. The association between staff composition and VMMC *unit cost* underscores the important potential role of task shifting from physicians to nurses in improving efficiency, especially since previous studies have demonstrated that such task shifting does not increase the numbers of adverse events reported [32].

Though our study provided some of the most comprehensive evidence to date on VMMC *total* and *unit cost* and their correlation with supply-side characteristics, a number of limitations should be kept in mind when considering our findings. Our study focused on one VMMC service delivery modality (fixed site) and one approach (surgical). We were unable to report costs of VMMCs delivered through outreach and mobile service delivery. We were also unable to provide insight into the cost of VMMCs performed using devices such as the PrePex or the Shang Ring [33]. We retrospectively collected information on inputs, costs, outputs, and time allocation, although never more than 12 months subsequent to the period studied. We used routine monitoring data to capture information on outputs, and the detail, quality, and completeness of these data varied. Because we were unable to disaggregate the circumcisions performed by client age, we were unable to assess the extent to which cost per circumcision varied by client age. Moreover, we did not ascertain whether “difficulty” of circumcisions done at VMMC clinics co-located at hospitals differed from circumcisions in primary care settings. Though data collection instruments were designed to capture cost data related to demand creation, these data were not available in facilities and demand creation is therefore excluded from our cost calculations. Data on waste disposal were not measured and a previous study illustrated that this can constitute an important cost component [34]. Because we only considered service provider costs, costs incurred by VMMC clients were not included. Above-facility costs were also excluded. An additional limitation is the way staff time was allocated. Though our estimates of providers working exclusively on VMMC constitute an improvement on self-report since they were derived triangulating information from multiple sources, our calculation of effort attributable to VMMC for non-dedicated staff likely contributed to an underestimate of time spent on VMMC since VMMC is likely to be one of the more complex outpatient procedures provided. Our data were cross sectional and our methods only allowed us to explore costs and their associations with supply-side factors at a given point in time. Finally, our analytical strategy of modeling unit cost as a function of supply-side characteristics departed from traditional economic cost analysis by including the total number of VMMC per year in both sides of the equation. This approach could produce a spurious negative relationship between unit cost and scale in the presence of measurement error in the number of annual circumcisions. However, we showed that the results on the determinants of *unit cost* variation were consistent with the estimates from our models of *total cost* as a function of quantities and prices–as per traditional cost function estimation. Some of the heterogeneity in VMMC *unit cost* we observed may be due to variation in service quality unmeasured in our study. However, it is unlikely that the magnitude of differences observed can be explained by differences in service quality alone. Rather, this cost variation suggests that substantial efficiency gains could be made in VMMC service delivery in all four countries.

## Conclusion

Our analyses suggest several ways to increase the efficiency of VMMC services. Options to increase efficiency of VMMC programs in the short term include intervening to rapidly improve the efficiency of outlier clinics or close them. Focusing the provision of services in high and medium yield sites when demand is high, focusing on task shifting from physicians to nurses, and taking advantage of efficiencies created by integrating HIV services are all possibilities that should be considered. In the longer term, reductions in VMMC *unit cost* are likely by increasing the volume of VMMC clients at facilities by implementing effective demand generation activities [35–39].

## Supporting information

S1 Fig Sample distribution by facility type. *Second-level hospitals and one tertiary-level hospital in South Africa.
(DOCX)

S2 Fig Monthly output levels by country (seasonality). (DOCX)

S1 Methods Description of sensitivity analysis. (DOCX)

S2 Methods Estimation of total annual staff costs. (DOCX)

S1 Table Variable definitions. (DOCX)

S2 Table Descriptive analysis of variables included in regression models. (DOCX)

S3 Table GLM regression models. **Dependent variable is the natural logarithm of the facility-level total cost of VMMC services (without outliers).** ARV = antiretroviral; HTC = HIV testing and counseling; PMTCT = prevention of mother-to-child transmission; VMMC = voluntary medical male circumcision. All models are adjusted by country dummies and staff hourly wage (prices). 95% confidence interval in parentheses. *** p<0.01, ** p<0.05, * p<0.1.^a^Reference category = Hospital. ^b^ Nine observations with missing values on the number of HTC or PMTCT clients were imputed using 90 observations with the linear regression model: HTC (or PMTCT) number of clients = *b*_0_ + *b*_1_staff + *b*_2_VMMC + *b*_3_ facility type + *b*_4_country + e. %Percentage change in total cost compared to the reference category. ^c^ Percentage change in *total cost* per 10% change in independent variable.
(DOCX)

S4 Table GLM regression models. **Dependent variable is the natural logarithm of the facility-level unit cost of VMMC services (without outliers).** ARV = antiretroviral; HTC = HIV testing and counseling; PMTCT = prevention of mother-to-child transmission; VMMC = voluntary medical male circumcision. All models are adjusted by country dummies and staff hourly wage (prices). 95% confidence interval in parentheses. *** p<0.01, ** p<0.05, * p<0.1. ^a^ Reference category = Hospital. ^b^ Nine observations with missing values on the number of HTC or PMTCT clients were imputed using 90 observations with the linear regression model: HTC (or PMTCT) number of clients = *b*_0_ + *b*_1_staff + *b*_2_VMMC + *b*_3_ facility type + *b*_4_country + e. % Percentage change in *unit cost* compared to the reference category. ^c^ Percentage change in *unit cost* per 10% change in independent variable.
(DOCX)

## Abbreviations

ART: antiretroviral therapy
HTC: HIV testing and counseling
GLM: generalized linear model
ORPHEA: Optimizing the Response in Prevention: HIV Efficiency in Africa
PMTCT: prevention of mother-to-child transmission
PPP: purchasing power parity
SSA: sub-Saharan Africa
VMMC: voluntary medical male circumcision
