# Supplementary figures and images for: Influence of supply-side factors on voluntary medical male circumcision costs in Kenya, Rwanda, South Africa, and Zambia
Source: PLoS One. 2018 Sep 13;13(9):e0203121. doi: 10.1371/journal.pone.0203121 (PMC6136711; doi:10.1371/journal.pone.0203121)

**S1 _FIG**

**
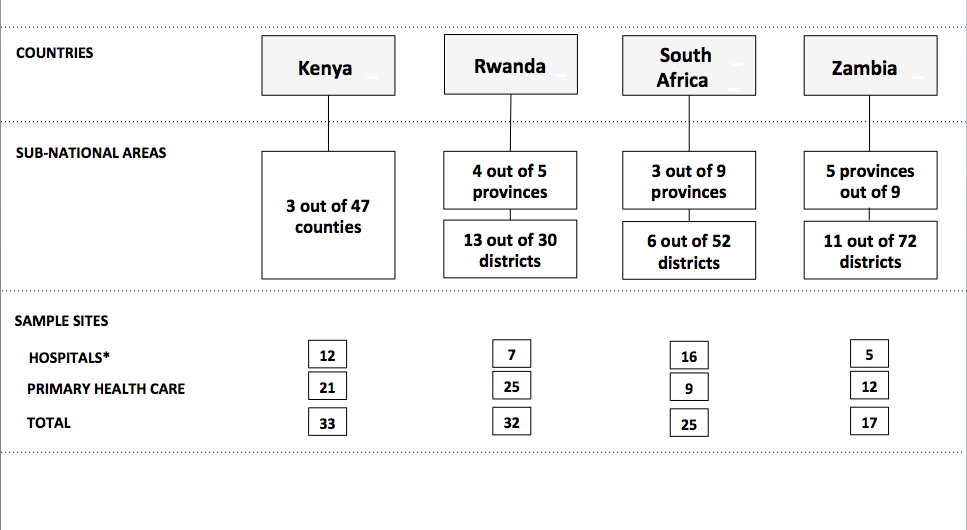
**

Supplement: S1 Fig — *Second-level hospitals and one tertiary-level hospital in South Africa. (DOCX) [file pone.0203121.s001.docx]

**S2_FIG**


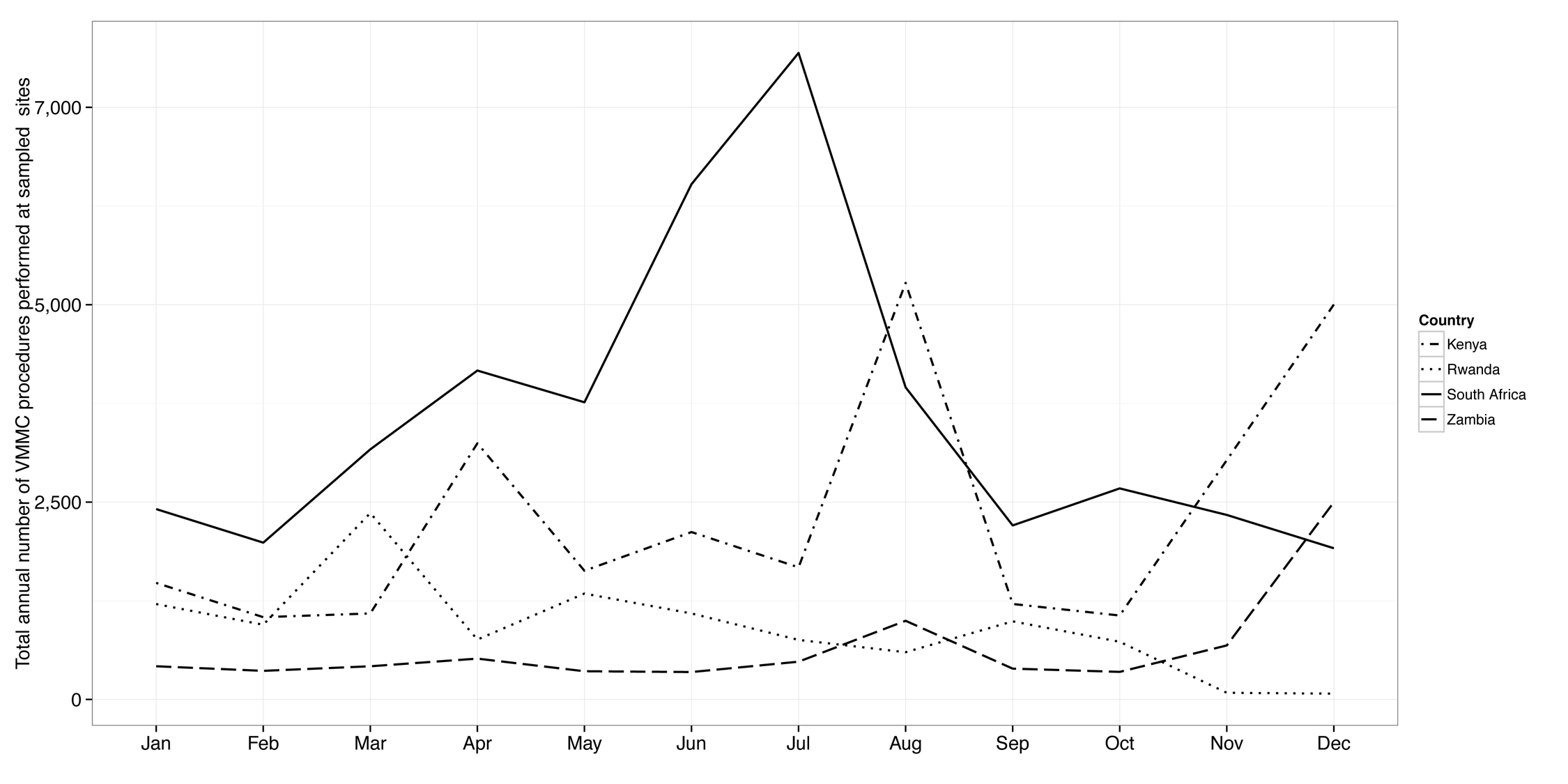

Supplement: S2 Fig — (DOCX) [file pone.0203121.s002.docx]
