# Supplementary material for: Influence of supply-side factors on voluntary medical male circumcision costs in Kenya, Rwanda, South Africa, and Zambia
Source: PLoS One. 2018 Sep 13;13(9):e0203121. doi: 10.1371/journal.pone.0203121 (PMC6136711; doi:10.1371/journal.pone.0203121)
Supplement: S1 Methods — (DOCX) [file pone.0203121.s003.docx]

**S1 Methods**

Because we used both data on the numbers of circumcisions performed during each month as well as the total number of procedures during the costing year, a probabilistic sensitivity analysis was performed to assess the uncertainty around the output variable (see Figure S4 and Table S5). We implemented the following steps iteratively. We first drew a random subset of 90% of the analytic sample. The output variable was then set as either the sum of the monthly output data or the total annual number of procedures reported, each with probability equal to 0.5. Finally, all costs calculations were performed anew. The number of iterations was defined heuristically until the standard error of the output variable decreased asymptotically (achieved at close to 1,000 iterations). These estimates did not vary significantly from our results.

**
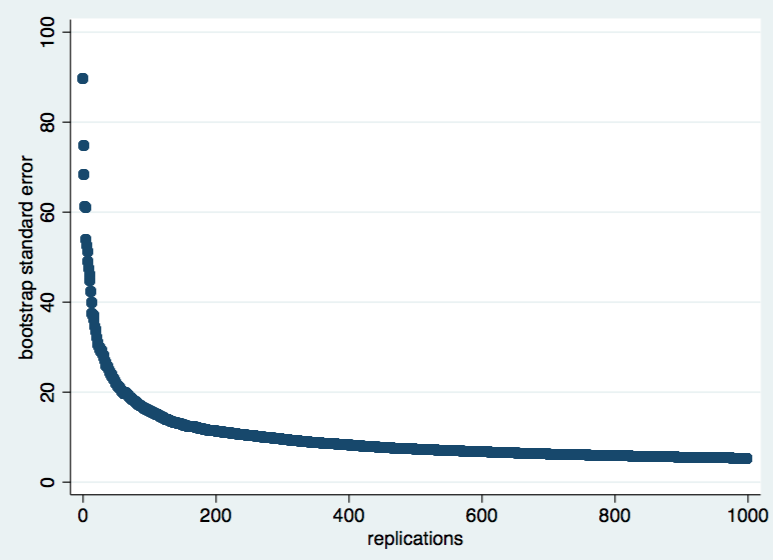
Optimal number of replications for the sensitivity analysis of VMMC costs**

**Sensitivity analysis of the VMMC cost including the variation of VMMC procedures reported**

|  |  | **VMMC cost** | |  | **VMMC clients** | |
| --- | --- | --- | --- | --- | --- | --- |
| **Country** | **N** | **Mean** | **p50** |  | **Mean** | **p50** |
|  |  |  |  |  |  |  |
| Kenya | 1000 | 68.7 | 43.9 |  | 853 | 648 |
| Rwanda | 1000 | 83.3 | 35.6 |  | 345 | 185 |
| South Africa | 1000 | 287.1 | 141.6 |  | 1513 | 887 |
| Zambia | 1000 | 81.8 | 41.1 |  | 460 | 228 |
|  |  |  |  |  |  |  |
| Total | 4000 | 130.2 | 65.6 |  | 793 | 487 |
