# Supplementary material for: Influence of supply-side factors on voluntary medical male circumcision costs in Kenya, Rwanda, South Africa, and Zambia
Source: PLoS One. 2018 Sep 13;13(9):e0203121. doi: 10.1371/journal.pone.0203121 (PMC6136711; doi:10.1371/journal.pone.0203121)
Supplement: S2 Methods — (DOCX) [file pone.0203121.s004.docx]

**S2 Methods**

To calculate staff costs for VMMC services, we estimated a measure of staff effort expended on VMMC service delivery by triangulating information related to time use obtained from program records, self-reports, field notes, and interviews with staff in charge of facilities. When providers reported working on VMMC only, their full effort was allocated to VMMC. In the case of non-dedicated providers (working on a combination of services including VMMC), effort attributable to VMMC was obtained by an estimate obtained from data on the combination of services provided (VMMC, HTC, PMTCT, and other), the annual number of VMMC clients in a facility, the number of other outpatient clients in a facility, and the average amount of time spent on VMMC and other services in the facility, as follows:

$\alpha_{i}=\frac{{VMMC clients}_{i}}{{Total facility clients}_{i}};\alpha\in(0,1]$ (1)

Annual VMMC staff costs were then obtained by multiplying full-time provider equivalents (i.e. number of weeks worked during the year and number of hours worked per week) times annual provider salaries, weighted by effort as follows:

${Annual staff costs}_{i}=\sum_{j=1}^{n} S_{j}\times\frac{h_{\mathrm{ij}}}{40}\times\frac{w_{\mathrm{ij}}}{52}\times\alpha_{\mathrm{ij}}^{k}$ (2)

where $i$ is an index for facilities; $j$ is an index for providers within facilities; $S_{j}$ is the average annual salary per staff cadre at the facility;$h_{j}$ denotes the average number of hours worked per week during the costing year, with a maximum of 40 working hours per week; $w_{j}$ represents the number of weeks worked during the costing year, with a maximum of 52 working weeks per year; $k=0$ if the provider is fully dedicated to VMMC and $k=1$ otherwise.
