# Supplementary material for: Influence of supply-side factors on voluntary medical male circumcision costs in Kenya, Rwanda, South Africa, and Zambia
Source: PLoS One. 2018 Sep 13;13(9):e0203121. doi: 10.1371/journal.pone.0203121 (PMC6136711; doi:10.1371/journal.pone.0203121)
Supplement: S1 Table — (DOCX) [file pone.0203121.s005.docx]

**S1 Table**

| **Variable categories and names** | **Description** | **Question asked in questionnaire** |
| --- | --- | --- |
| *Scale* |  |  |
| Annual number of MC clients | Natural log of number of VMMC clients per year | What was the number of pre-MC and circumcision procedures in the last month, during the entire calendar year, and for every month of the calendar year? |
| *Facility characteristics* |  |  |
| Primary health care facility | Indicator variable for level of service provision, dichotomized into hospitals (reference category) and primary health care clinics | Type of facility |
| *Community involvement* |  |  |
| Outreach | Variable for community outreach, dichotomized into community promotion activities undertaken or not undertaken (reference category) | Does this facility refer/offer:  MC promotional activities, such as fliers, pamphlets, leaflets (specify) |
| *Staff composition* |  |  |
| Task shifting | Variable for task shifting, dichotomized into involvement of physicians in the provision of VMMC (reference category) and delegation of tasks to less specialized health workers | Proportion of FTE of medical doctors divided by FTE of other health staff that provide MC services. |
| Average staff experience (in years) | Average number of years of professional experience | How long (in years) has [each staff] been working at this facility? |
| *Scope* |  |  |
| Annual number of PMTCT clients (ln) | Natural log of number of PMTCT clients per year | What was the number of antenatal clients tested in the last month, during the entire calendar year, and for every month of the calendar year? |
| Annual number of HTC clients (ln) | Natural log of number of HTC clients per year | What was the number of individuals attending HTC in the last month, during the entire calendar year, and for every month of the calendar year? |
| Facility provides ART | Facility that provides antiretroviral therapy services | \| Are antiretroviral therapy (ART) services provided at this site? \| \| --- \| \| |
| *Location* |  |  |
| Countries | Indicator variables for countries, including Kenya (reference category), Rwanda, South Africa, Zambia |  |
| *Input prices* |  |  |
| Medical doctor hourly wage | Salary/FTE of the medical doctors working on HIV services | Hours worked multiplied by hour staff rate divided by FTE for medical doctors |
| Nurse hourly wage | Salary/FTE of the nurses working on HIV services | Hours worked multiplied by hour staff rate divided by FTE for specialized nurses |
| Registered nurse hourly wage | Salary/FTE of the registered nurses working on HIV services | Hours worked multiplied by hour staff rate divided by FTE for registered nurses |
| Other health staff hourly wage | Salary/FTE of other health staff working on HIV services | Hours worked multiplied by hour staff rate divided by FTE for other health staff |
| Indirect staff hourly wage | Salary/FTE of the indirect staff working on HIV services | Hours worked multiplied by hour staff rate divided by FTE for indirect staff |
