# Supplementary material for: Influence of supply-side factors on voluntary medical male circumcision costs in Kenya, Rwanda, South Africa, and Zambia
Source: PLoS One. 2018 Sep 13;13(9):e0203121. doi: 10.1371/journal.pone.0203121 (PMC6136711; doi:10.1371/journal.pone.0203121)
Supplement: S2 Table — (DOCX) [file pone.0203121.s006.docx]

**S2 Table**

|  | Kenya | | | |  | Rwanda | | | |  | South Africa | | | |  | Zambia | | | |  | Total | | | |
| --- | --- | --- | --- | --- | --- | --- | --- | --- | --- | --- | --- | --- | --- | --- | --- | --- | --- | --- | --- | --- | --- | --- | --- | --- |
|  | N | Mean | SD | Median |  | N | Mean | SD | Median |  | N | Mean | SD | Median |  | N | Mean | SD | Median |  | N | Mean | SD | Median |
| Natural logarithm of the Average cost per VMMC | 31 | 6.5 | 0.8 | 6.5 |  | 29 | 5.3 | 1.0 | 5.1 |  | 24 | 6.9 | 1.0 | 6.9 |  | 16 | 5.6 | 1.2 | 5.4 |  | 100 | 6.1 | 1.2 | 6.3 |
| ***Scale*** |  |  |  |  |  |  |  |  |  |  |  |  |  |  |  |  |  |  |  |  |  |  |  |  |
| Annual VMMCs performed (100s) | 31 | 9.1 | 8.1 | 6.9 |  | 29 | 3.1 | 3.6 | 1.6 |  | 24 | 17.5 | 24.9 | 9.7 |  | 16 | 4.9 | 5.4 | 2.3 |  | 100 | 8.7 | 14.2 | 5.7 |
| ***Community involvement*** |  |  |  |  |  |  |  |  |  |  |  |  |  |  |  |  |  |  |  |  |  |  |  |  |
| Outreach | 31 | 74% | 44% | 100% |  | 29 | 21% | 41% | 0% |  | 24 | 54% | 51% | 100% |  | 16 | 81% | 40% | 100% |  | 100 | 55% | 50% | 100% |
| ***Staff composition*** |  |  |  |  |  |  |  |  |  |  |  |  |  |  |  |  |  |  |  |  |  |  |  |  |
| Task shifting | 31 | 29% | 46% | 0% |  | 29 | 90% | 31% | 100% |  | 24 | 38% | 49% | 0% |  | 16 | 81% | 40% | 100% |  | 100 | 57% | 50% | 100% |
| Medical doctor hourly wage | 31 | 6.5 | 2.7 | 6.8 |  | 29 | 12.0 | 0.3 | 12.0 |  | 24 | 52.2 | 18.5 | 50.5 |  | 16 | 7.8 | 0.9 | 7.8 |  | 100 | 19.3 | 20.8 | 12.0 |
| Nurse hourly wage | 31 | 5.4 | 1.4 | 5.5 |  | 29 | 5.6 | 4.0 | 4.8 |  | 24 | 30.3 | 27.6 | 23.5 |  | 16 | 8.9 | 3.5 | 8.7 |  | 100 | 12.0 | 17.1 | 5.5 |
| Registered nurse hourly wage | 31 | 3.9 | 1.9 | 3.8 |  | 29 | 2.8 | 0.4 | 2.8 |  | 24 | 16.8 | 15.4 | 13.2 |  | 16 | 8.8 | 4.7 | 8.3 |  | 100 | 7.5 | 9.6 | 4.1 |
| Other health staff hourly wage | 31 | 3.3 | 1.6 | 3.6 |  | 29 | 3.6 | 1.9 | 3.3 |  | 24 | 7.6 | 8.9 | 5.8 |  | 16 | 3.7 | 2.6 | 3.5 |  | 100 | 4.5 | 4.9 | 3.3 |
| Indirect staff hourly wage | 31 | 3.0 | 1.4 | 3.5 |  | 29 | 4.3 | 1.9 | 3.9 |  | 24 | 11.4 | 13.8 | 6.8 |  | 16 | 3.4 | 1.6 | 3.0 |  | 100 | 5.5 | 7.6 | 3.9 |
| Years of professional experience | 31 | 3.4 | 2.7 | 3.0 |  | 29 | 4.6 | 1.9 | 4.1 |  | 24 | 7.1 | 3.3 | 7.3 |  | 16 | 5.9 | 2.4 | 4.8 |  | 100 | 5.0 | 2.9 | 4.5 |
| ***Scope*** |  |  |  |  |  |  |  |  |  |  |  |  |  |  |  |  |  |  |  |  |  |  |  |  |
| Annual number of HTC clients (ln) | 31 | 7.4 | 1.1 | 7.4 |  | 29 | 6.5 | 3.5 | 7.9 |  | 24 | 8.5 | 0.8 | 8.4 |  | 16 | 7.3 | 1.2 | 7.5 |  | 100 | 7.4 | 2.2 | 7.8 |
| Annual number of PMTCT clients (ln) | 31 | 6.3 | 2.1 | 6.8 |  | 29 | 4.9 | 2.8 | 6.2 |  | 24 | 6.2 | 2.4 | 6.9 |  | 16 | 7.5 | 1.0 | 7.6 |  | 100 | 6.1 | 2.4 | 6.7 |
| ART | 31 | 10% | 30% | 0% |  | 29 | 0% | 0% | 0% |  | 24 | 4% | 20% | 0% |  | 16 | 31% | 48% | 0% |  | 100 | 9% | 29% | 0% |
