# Supplementary material for: Influence of supply-side factors on voluntary medical male circumcision costs in Kenya, Rwanda, South Africa, and Zambia
Source: PLoS One. 2018 Sep 13;13(9):e0203121. doi: 10.1371/journal.pone.0203121 (PMC6136711; doi:10.1371/journal.pone.0203121)
Supplement: S3 Table — Dependent variable is the natural logarithm of the facility-level total cost of VMMC services (without outliers). ARV = antiretroviral; HTC = HIV testing and counseling; PMTCT = prevention of mother-to-child transmission; VMMC = voluntary medical male circumcision. All models are adjusted by country dummies and staff hourly wage (prices). 95% confidence interval in parentheses. *** p<0.01, ** p<0.05, * p<0.1.aReference category = Hospital. b Nine observations with missing values on the number of HTC or PMTCT clients were imputed using 90 observations with the linear regression model: HTC (or PMTCT) number of clients = b0 + b1staff + b2VMMC + b3 facility type + b4country + e. %Percentage change in total cost compared to the reference category. c Percentage change in total cost per 10% change in independent variable. (DOCX) [file pone.0203121.s007.docx]

**S3 Table**

|  | Specification (1)^b^ | % | Specification (2)^b^ | % |
| --- | --- | --- | --- | --- |
| Annual number of VMMC clients (ln) | 0.699*** | 7 ^c^ | 1.595*** | 16 ^c^ |
|  | (0.583 - 0.814) |  | (0.769 - 2.420) |  |
| Square of annual number of VMMC clients (ln) |  |  | -0.073** | -1 ^c^ |
|  |  |  | (-0.139 - -0.006) |  |
| Primary health care facility^a^ | -0.286** | -25 | -0.275* | -24 |
|  | (-0.571 - -0.000) |  | (-0.554 - 0.005) |  |
| Average staff experience (in years) | 0.214** | 24 | 0.193** | 21 |
|  | (0.041 - 0.388) |  | (0.023 - 0.364) |  |
| Square of average staff experience (in years) | -0.014** | -1 | -0.013** | -1 |
|  | (-0.027 - -0.002) |  | (-0.026 - -0.001) |  |
| Outreach | 0.308** | 36 | 0.326** | 39 |
|  | (0.041 - 0.575) |  | (0.064 - 0.588) |  |
| Task shifting | -0.552*** | -42 | -0.556*** | -43 |
|  | (-0.833 - -0.271) |  | (-0.831 - -0.281) |  |
| Annual number of HTC clients (ln) | -0.061 | -1 ^c^ | -0.046 | 0 ^c^ |
|  | (-0.136 - 0.014) |  | (-0.121 - 0.028) |  |
| Annual number of PMTCT clients (ln) | -0.085** | -1 ^c^ | -0.092** | -1 ^c^ |
|  | (-0.161 - -0.008) |  | (-0.167 - -0.018) |  |
| Facility provides ART | -0.505** | -40 | -0.460* | -37 |
|  | (-0.976 - -0.034) |  | (-0.923 - 0.002) |  |
| Constant | 6.339*** |  | 3.590*** |  |
|  | (5.304 - 7.374) |  | (0.884 - 6.296) |  |
| Observations | 97 |  | 97 |  |
