# Supplementary material for: Influence of supply-side factors on voluntary medical male circumcision costs in Kenya, Rwanda, South Africa, and Zambia
Source: PLoS One. 2018 Sep 13;13(9):e0203121. doi: 10.1371/journal.pone.0203121 (PMC6136711; doi:10.1371/journal.pone.0203121)
Supplement: S4 Table — Dependent variable is the natural logarithm of the facility-level unit cost of VMMC services (without outliers). ARV = antiretroviral; HTC = HIV testing and counseling; PMTCT = prevention of mother-to-child transmission; VMMC = voluntary medical male circumcision. All models are adjusted by country dummies and staff hourly wage (prices). 95% confidence interval in parentheses. *** p<0.01, ** p<0.05, * p<0.1. a Reference category = Hospital. b Nine observations with missing values on the number of HTC or PMTCT clients were imputed using 90 observations with the linear regression model: HTC (or PMTCT) number of clients = b0 + b1staff + b2VMMC + b3 facility type + b4country + e. % Percentage change in unit cost compared to the reference category. c Percentage change in unit cost per 10% change in independent variable. (DOCX) [file pone.0203121.s008.docx]

**S4 Table**

|  | Specification (1)^b^ | % | Specification (2)^b^ | % |
| --- | --- | --- | --- | --- |
| Annual number of VMMC clients (ln) | -0.300*** | -3 ^c^ | 0.584 | 6 ^c^ |
|  | (-0.415 - -0.184) |  | (-0.226 - 1.395) |  |
| Square of annual number of VMMC clients (ln) |  |  | -0.072** | -1 ^c^ |
|  |  |  | (-0.137 - -0.007) |  |
| Primary health care facility^a^ | -0.305** | -26 | -0.286** | -25 |
|  | (-0.589 - -0.022) |  | (-0.565 - -0.008) |  |
| Average staff experience (in years) | 0.226*** | 25 | 0.204** | 23 |
|  | (0.056 - 0.397) |  | (0.035 - 0.372) |  |
| Square of average staff experience (in years) | -0.015** | -1 | -0.014** | -1 |
|  | (-0.028 - -0.002) |  | (-0.026 - -0.001) |  |
| Outreach | 0.239* | 27 | 0.262** | 30 |
|  | (-0.011 - 0.489) |  | (0.016 - 0.507) |  |
| Task shifting | -0.595*** | -45 | -0.591*** | -45 |
|  | (-0.869 - -0.320) |  | (-0.860 - -0.323) |  |
| Annual number of HTC clients (ln) | -0.065* | -1 ^c^ | -0.049 | 0 ^c^ |
|  | (-0.140 - 0.010) |  | (-0.124 - 0.026) |  |
| Annual number of PMTCT clients (ln) | -0.080** | -1 ^c^ | -0.089** | -1 ^c^ |
|  | (-0.156 - -0.004) |  | (-0.164 - -0.014) |  |
| Facility provides ART | -0.528** | -41 | -0.481** | -38 |
|  | (-0.998 - -0.058) |  | (-0.943 - -0.019) |  |
| Constant | 6.467*** |  | 3.750*** |  |
|  | (5.442 - 7.492) |  | (1.087 - 6.413) |  |
| Observations | 97 |  | 97 |  |
